# Improving health outcomes through concurrent HIV program scale-up and health system development in Rwanda: 20 years of experience

**DOI:** 10.1186/s12916-015-0443-z

**Published:** 2015-09-09

**Authors:** Sabin Nsanzimana, Krishna Prabhu, Haley McDermott, Etienne Karita, Jamie I. Forrest, Peter Drobac, Paul Farmer, Edward J. Mills, Agnes Binagwaho

**Affiliations:** Institute of HIV Disease Prevention and Control, Rwanda Biomedical Centre, Kigali, Rwanda; Harvard University Medical School, Boston, USA; Partners in Health, Boston, USA; School of Population and Public Health, University of British Columbia, Vancouver, Canada; Global Evaluative Sciences, Vancouver, Canada; Ministry of Health of Rwanda, Kigali, Rwanda; Geisel School of Medicine, Dartmouth College, Hanover, USA; Project San Francisco, Kigali, Rwanda; Basel Institute for Clinical Epidemiology & Biostatistics and Swiss Tropical and Public Health institute, University of Basel, Basel, Switzerland

**Keywords:** Antiretroviral therapy, HIV/AIDS, Rwanda, Sub-Saharan Africa, Universal healthcare

## Abstract

The 1994 genocide against the Tutsi destroyed the health system in Rwanda. It is impressive that a small country like Rwanda has advanced its health system to the point of now offering near universal health insurance coverage. Through a series of strategic structural changes to its health system, catalyzed through international assistance, Rwanda has demonstrated a commitment towards improving patient and population health indicators. In particular, the rapid scale up of antiretroviral therapy (ART) has become a great success story for Rwanda. The country achieved universal coverage of ART at a CD4 cell count of 200 cells/mm^3^ in 2007 and increased the threshold for initiation of ART to ≤350 cells/mm^3^ in 2008. Further, 2013 guidelines raised the threshold for initiation to ≤500 cells/mm^3^ and suggest immediate therapy for key affected populations. In 2015, guidelines recommend offering immediate treatment to all patients. By reviewing the history of HIV and the scale-up of treatment delivery in Rwanda since the genocide, this paper highlights some of the key innovations of the Government of Rwanda and demonstrates the ways in which the national response to the HIV epidemic has catalyzed the implementation of interventions that have helped strengthen the overall health system.

## Background

Rwanda is a landlocked country in the Great Lakes region of East Africa, bordered by Uganda, Tanzania, Burundi, and the Democratic Republic of the Congo. It has a predominantly dense, but mainly rural population and the average age of Rwandans is 22.7 years [1]. The population of Rwanda has grown at a rate of 2.6 % every year from 2002 to 2012 and is expected to reach 13.3 million by 2022.

On July 1, 1962, Rwanda was granted full political independence from Belgium, following colonial rule since 1923. In the years following independence, the Government of Rwanda, dominated by Hutu extremists, began systematically oppressing the Tutsi minority population. In April of 1994, in a terror that lasted 100 days, Rwanda’s infrastructure and human resources were catastrophically damaged by a genocide that claimed the lives of more than 1,000,000 Tutsis and moderate Hutus. When the genocide ended, a new government began the difficult process of returning peace, security, and stability to the country.

In 2005, in combination with many efforts to reclaim Rwanda’s prosperity, the Government of Rwanda began reforming operations, including redistricting internal geopolitical boundaries and a decentralization of governance systems, such as the health sector. These reforms now define the five provinces, with 30 districts per province. These are further subdivided into sectors, each containing 14,953 *umudugus* (villages) of approximately 50 to 100 households. This intentional, structural organization of Rwanda has helped the country achieve greater decentralization and localization of health care in a way that has substantially improved its population health. Rwanda has among the best population health indicators in the region and the country has almost met each of the health-related Millennium Development Goals [2, 3]. More than 97 % of Rwandan infants are vaccinated against ten different diseases and 69 % of births are attended to by trained clinicians at health facilities [2, 3]. Premature mortality rates have fallen precipitously in recent years, and life expectancy has almost doubled since the end of the genocide in 1994 [4].

**Fig. 1:**
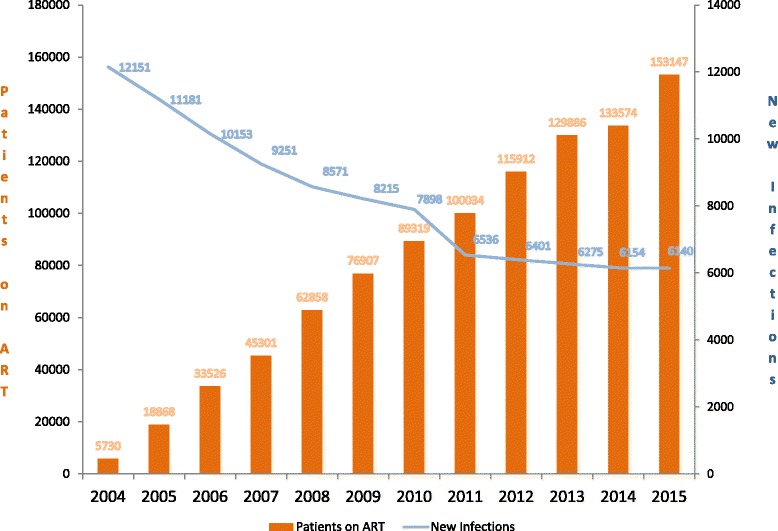
Decline in new HIV infections (blue line) and increasing coverage of antiretroviral therapy (ART) in Rwanda, 2004–2015. Source: Institute of HIV/AIDS Disease Prevention & Control, Rwanda Biomedical Centre; reproduced with permission

The strengthening of the health sector in the 20 years since the end of the genocide is tightly linked to Rwanda’s response to the HIV epidemic. Through a series of strategic decisions, the formation of strong partnerships and global mobilization of resources, Rwanda has made remarkable progress at scaling up access to antiretroviral therapy (ART) and improving the delivery of care and support to an estimated 204,899 people living with HIV in the country [5]. Prior to 2002, there were less than 100 people on ART. Today, more than 150,000 patients are on treatment (Fig. 1). This scale-up occurred by incrementally raising the CD4 threshold for access to treatment. Rwanda first achieved universal coverage of ART at a CD4 cell count threshold of 200 cells/mm^3^ in 2007, increased the threshold to ≤350 cells/mm^3^ in 2008, and 2013 guidelines raised it to ≤500 cells/mm^3^, with exceptions for immediate therapy for key populations. In 2015, guidelines have recommended offering immediate treatment to all patients regardless of CD4 eligibility. It is through this scale-up that we describe some of the key innovations in the health system in the previous two decades and demonstrate how these innovations have helped strengthen the overall population health of the country.

### HIV and the genocide against Tutsis in Rwanda, 1994

HIV was first reported in Rwanda in 1983 by a team of Belgian scientists. In 1986, the nation conducted its first population-based sero-prevalence survey that reported an urban prevalence of 18 % and a rural prevalence of 1 % [6]. For nearly a decade after, there was little awareness of HIV/AIDS, and most physicians were unable to recognize, diagnose, or treat the condition. People living with HIV were kept in isolation wards with poor sanitary conditions and without safety precautions to prevent transmission between patients and hospital staff. There was little to no access to HIV treatment in Rwanda prior to 1994.

The 1994 genocide profoundly set back all of Rwanda’s development efforts. In the wake of the 100-days of killing, two million were left homeless and the health system had collapsed [7]. Rape, as a weapon of war, was used against more than 250,000 women and helped fuel a sharp increase in HIV infections after the genocide [8]. Large population migrations in and out of Rwanda in the years that followed the genocide made it difficult for authorities to detect and control the spread of new infections [9]. The genocide led to a ruin of physical health infrastructure (hospitals, clinics, public health labs, etc.) and generated a massive exodus of skilled medical personnel. By the end of the genocide, nearly 80 % of physicians had been killed or had fled the country [10]. Fewer than ten pediatricians were practicing in all of Rwanda in the year that followed. It was an event that had a profound effect on the identity of the nation and its response to HIV since 1994 has been a demonstration of the resiliency of humanity.

### Approaching the HIV epidemic after the genocide, 1996–2002

In 1995, the National Program for HIV/AIDS Control (PNLS) was re-established with a renewed mission to control the AIDS epidemic in Rwanda. The initial goal of the PNLS was to educate Rwandans about how to prevent HIV infection. The campaign first gained momentum when President Kagame spontaneously attended a PNLS conference, voicing his support for the battle against HIV/AIDS and declaring it a top priority for his administration. However, like many sub-Saharan African countries at the time, limited government resources with limited foreign assistance made the high cost of HIV treatment prohibitively out of reach. In 1999, treatment costs were as high as US$ 6,065 per patient per year, and antiretroviral prices would account for 92 % of the total cost of care. Only 202 people living with HIV in Rwanda at this time were able to afford the out-of-pocket expense of purchasing ART on a global market [11].

By 1999, the Ministry of Health had begun expanding HIV testing facilities and laboratory capacity to prepare for expanding access to ART. In early 2000, the universal treatment program began with a small government created fund to offer free ART at the Kigali Teaching Hospital. Meanwhile, medical practitioners had begun intensive HIV clinical and administrative training within and outside Rwanda. Training also helped improve record keeping and reporting practices. By the early 2000s, HIV program scale-up was ready to take shape, but funding remained a limiting factor.

### Funding, scale-up, decentralization, and the integration of programs 2002–2007

In 2002, two major events catalyzed the scale-up of Rwanda’s HIV program. First, the Ministry of Health disseminated standardized, national protocols that gave medical centers, district hospitals, and referral centers authorization and instructions for providing care to people living with HIV. Protocols were drafted based on similar documents written by the United States Centers for Disease Control and focused on training healthcare providers to perform diagnoses and facilitate patient retention through follow-up. Second, major sources of funding, beginning in 2002, helped accelerate the expansion of HIV services. Initial funding came from the World Bank in the form of a US$ 30.5 million 3-year award to scale-up access to testing, mitigate the social impact of HIV, and purchase ART for those in need. This was closely followed by the Global Fund to Fight AIDS, Tuberculosis and Malaria, awarding Rwanda US$ 34 million to further strengthen health facility capacity for testing and treatment and to scale-up prevention of mother-to-child transmission (PMTCT) services. The same year, the US President’s Emergency Plan for AIDS Relief (PEPFAR) was launched and granted Rwanda US$ 39 million to support increased access to ART and PMTCT programs.

The influx of funding in 2002, delivered by a multitude of partnerships with NGOs and international donors, occurred in the context of a weaker government capacity. The proliferation of NGO involvement in Rwanda often led to a duplication of services, stemming from poor coordination between the government and the many independent agencies providing care. Each partner used different approaches and reporting channels and there was limited enforcement of the strategies created under PNLS. Some implementing partners reported directly to their donors without providing any information to the Government of Rwanda. This severely limited access to critical information such as data on epidemiological trends to monitor and inform the progress of the national response. This led to the development of a technical coordination team in 2005 that aimed to coordinate decision-making and draft new guidelines and protocols for HIV service delivery in Rwanda. The group was led by the PNLS and included representatives from US government organizations (CDC, PEPFAR, USAID) and UN partners (WHO, UNICEF, UNAIDS), as well as faith-based organizations and local NGOs. The team was charged with the formidable task of promoting the integration of funds and services, expanding the geographical reach of programs, increasing health worker performance, improving financial accessibility and accountability, improving overall awareness of HIV, and decreasing stigma and discrimination.

**Fig. 2:**
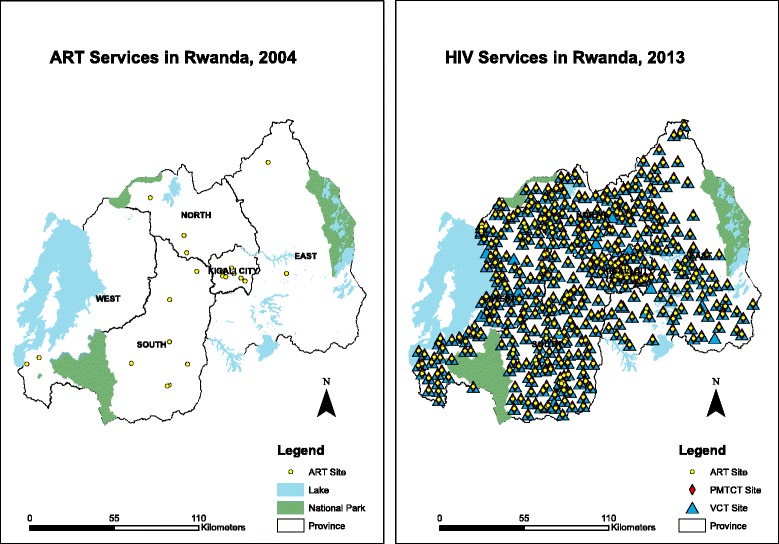
The decentralization of HIV services depicted by the number and location of services in Rwanda in 2004 (left) and in 2013 (right). Source: Institute of HIV/AIDS Disease Prevention & Control, Rwanda Biomedical Centre; reproduced with permission

Over the next several years, the coordination of HIV services in Rwanda was scaled-up by focusing on the decentralization of care. This is depicted in Fig. 2, which shows the number and location of HIV services in Rwanda in 2004 on the left and in 2013 on the right. As of 2013, more than 465 health facilities now provide HIV services, including delivery of ART [5]. This was achieved by making training investments in health facilities, strategic decisions on the supply chain of ART across the country, and continuously reviewing HIV protocols to keep up to date with global treatment guidelines. Patient and pharmacy files were standardized and reporting for routine monitoring and evaluation was made easier for local health facilities. This included the development of a standardized web-based electronic reporting system called TRACnet, launched in 2005 and which replaced the paper-based facility-level reporting system with one-way mobile phone technology that sent a standardized set of monthly health indicators to a centralized database in Kigali. Today, the TRACnet database has continued to permit real-time monitoring and evaluation of the national HIV care program, providing empirical evidence for the cumulative number of people on ART and the rate of new diagnoses (Fig. 1).

### Supporting health system innovations

There were a number of other secondary health system innovations that also helped strengthen Rwanda’s HIV response. Some international donors, particularly the Global Fund, were more open to funding more non-disease specific interventions that indirectly helped foster the scale-up of the HIV program, and improve overall population health. Two notable health system interventions that helped achieve this were the *Multelles de santé*, a community-based mutual insurance scheme, and the implementation of a performance-based health financing (PBF) scheme [12].

The high financial burden of user fees led to an overall decline in per-capita visits to health care facilities between 1997 and 1999 [13]. The inaccessibility of health services to the poor led implementers in Rwanda to test a pre-payment, community-based mutual insurance scheme in three districts of Rwanda, namely the *Mutuelles.* The Government of Rwanda began implementing the *Mutuelles* in 1999 to provide affordable basic services, especially child and maternal care, to the uninsured population. Over the next 7 years, the program was brought to scale and solidified into law in 2008. Approximately half of funding for the *Mutuelles* comes from annual member premiums. The remaining half is obtained through transfers from other insurance funds, charitable organizations, NGOs, development partners, and the Government of Rwanda. In particular, the Global Fund to Fight AIDS, Tuberculosis and Malaria funded the annual premium costs for the poorest 16 % of the population [14]. An impact evaluation of the *Mutuelles* has shown an increase in the utilization of healthcare and a decrease in catastrophic health spending among its members [15]. The *Mutuelles* program has successfully demonstrated that eliminating financial barriers to healthcare increases health service utilization and improves population-level health outcomes for all, including people living with HIV [14, 15].

The second major health system innovation was the implementation of PBF in 2001. The Government of Rwanda partnered with two NGOs and the School of Public Health at the National University of Rwanda to experiment with the implementation of PBF. The PBF program provided payments to health workers to incentivize high-quality care and, in 2006, the program was scaled-up across the country [16]. Incentives were distributed to health facilities based on the facility’s quality performance measures. The HIV program particularly benefited from the implementation of PBF, since payments were given for several key HIV care indicators, including the number of new adults and infants on ART, the number of HIV-positive pregnant mothers put on ART during pregnancy, and the number of HIV patients who receive a CD4 test in accordance with national guidelines. The program has been shown to have an effect on several health outcomes and it continues to be empirically evaluated and discussed in the literature [16, 17].

### Financial sustainability and human resource challenges, 2008 to present

In 2011, existing HIV coordinating mechanisms were dissolved and restructured into the Rwanda Biomedical Center to facilitate better integration with other disease-specific programs and create operational efficiencies that would generate more value in the face of declining resources. Rwanda was among the first countries to dissolve its national AIDS control commission in favor of a more integrated approach. By 2012, 97 % of all health facilities offered voluntary counseling and testing services, 97 % of all health facilities offered PMTCT services, and 89 % of all health facilities offered ART [18]. This has translated into impressive health outcomes for people living with HIV in Rwanda, including increased life expectancy [19] and high rates of retention in care [20, 21].

**Fig. 3:**
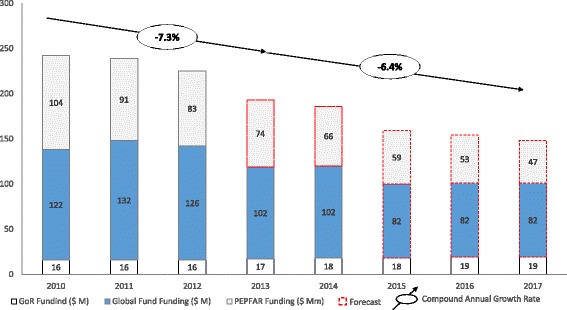
Funding changes since 2010 and predictions for future funding challenges for the HIV program in Rwanda. Source: Institute of HIV/AIDS Disease Prevention & Control, Rwanda Biomedical Centre; reproduced with permission

Yet, despite the many successes in Rwanda’s response to HIV, these health system gains remain fragile. As external donor funding for HIV programs has continued to decline at a rapid rate, the need for new resources to support programming has become increasingly crucial. By 2014, the annual cost of sustaining Rwanda’s HIV programs had grown to nearly US$ 200 million. This comprehensive budget, that supports HIV prevention, treatment, and control programming, was 80 % funded by external supporters. An overall decline in foreign assistance means the government must look to new ways to innovate its health system in an effort to improve financial sustainability while not compromising gains made in health outcomes. This challenge is depicted in Fig. 3, which shows the estimated funding gap for supporting the HIV program in Rwanda in future years.

In an effort to address the substantial burden of labor costs in the HIV program, the Rwanda developed new policies for the implementation of human resource task shifting in 2010. The program to train more than 500 nurses to deliver HIV care, including prescribing ART, has achieved high levels of retention and improved patient health outcomes [22]. The task shifting momentum also catalyzed the training of more than 45,000 community healthcare workers in Rwanda. This program has been successfully demonstrated to further improve patient retention in care, treatment, and support and minimize loss to follow-up [21]. Since 2012, these programs have been strengthened by the Human Resources for Health Program, focusing on knowledge transfer, sustained collaboration, and the establishment of a new medical residency, nursing specialty, health management, and oral health programs within the Rwandan education system [23]. As Rwanda works to become more financially sustainable in the delivery of HIV program services, strengthening human resources and maximizing efficiencies will be a key component of its strategy. Task shifting in other clinical areas and integrating these clinical areas within the existing training environment in the field of HIV can help achieve greater overall health system development.

### Conclusions and lessons learned

Rwanda has made remarkable development progress in the 20 years since the end of the genocide against the Tutsis. A strong commitment to an integrated and evidence-based response to the HIV epidemic has fostered substantial improvements in health outcomes for all Rwandans. Many countries in sub-Saharan Africa are also looking to innovative ways address their own HIV epidemics. The evolution of HIV program scale-up in Rwanda should teach us that strong leadership and the investment of resources in programs that strengthen the health system as a whole are some of the architectural features of health development in Rwanda that are laudable. The small country with a population that also speaks the same language has fostered the decentralization of services during their scale-up, which has also contributed to Rwanda’s overall success. Some aspects of the health systems innovations described in this paper are transferable to other countries; others are not. However, common to all sub-Saharan African countries, is a crucial need to find new efficiencies through better integration of delivery systems, revenue and funding streams, and local, regional, and global cooperation to continue improving the delivery of HIV program services.

## Abbreviations

ART: Antiretroviral therapy
PBF: Performance-based financing
PEPFAR: US President’s Emergency Plan for AIDS Relief
PMTCT: Prevention of mother-to-child transmission
PNLS: National Program for HIV/AIDS Control
